# Peer Review of “AI-Driven and Automated Systems for Continuous Oxygen Saturation Monitoring in Long-Term Oxygen Therapy: Systematic Review”

**DOI:** 10.2196/111437

**Published:** 2026-10-07

**Authors:** Juhee Lee

**Affiliations:** 1 Yonsei University College of Nursing, Seoul, Republic of Korea

**Keywords:** long-term oxygen therapy, artificial intelligence, machine learning, oxygen saturation monitoring, pulse oximetry, closed-loop systems, automated oxygen titration, motion artifacts, chronic obstructive pulmonary disease, systematic review

*This is a peer-review report for “AI-Driven and Automated Systems for Continuous Oxygen Saturation Monitoring in Long-Term Oxygen Therapy: Systematic Review.”*

## Round 1 Review

### Specific Comments

#### Major Comments

This paper [1] aims to evaluate AI-based and automated systems for long-term oxygen therapy (LTOT), addressing a timely and clinically relevant topic given the rapid advancement in personalized and responsive respiratory care technologies. The systematic approach and effort to integrate performance and usability dimensions are commendable. However, in its current form, the manuscript requires substantial revisions before it can be considered for publication. In particular, I would like to highlight the following concerns.

1. Lack of clearly defined research goal and review question.

The research goal and review question are not clearly described. This makes it difficult to understand whether the review is meant to summarize existing technologies, compare the performance of AI and automated systems, or evaluate how these systems can be applied in real clinical settings.

2. Insufficient background on technological context and rationale.

In the Introduction section, the paper should provide more background to help readers understand why the new approach is important. It should briefly explain how LTOT technology has changed over time, what makes AI-based systems different, and why this review is timely and needed. While the Introduction mentions some clinical problems that AI aims to solve—such as inefficiency of fixed flow and poor response to activity changes—it does not explain how older technologies tried to handle these issues or where they fell short.

3. Misalignment between stated objectives and inclusion criteria.

Although the Introduction emphasizes the need for a comprehensive review of AI technologies in the context of LTOT, the actual inclusion criteria are limited to studies addressing technical challenges such as motion-induced signal distortions (eg, convolutional neural network–based denoising, wavelet transforms), low-perfusion signal management, mitigation of skin tone–related measurement bias, and signal stability over 24 hours.

4. Unclear populations in reviewed studies.

The manuscript does not clearly describe which patient populations were included in the evaluation of AI or automated systems. Without this information, it is difficult to assess the clinical relevance and generalizability of the findings. Given that LTOT is most commonly used in older adult or chronic obstructive pulmonary disease populations, it is essential to specify whether the reviewed studies included such groups, especially in real-world or home-care settings.

#### Minor Comments

1. Please consider expanding the summary table to include more descriptive information (for example, publication year and country, participant characteristics, and study design). Also, to reduce confusion, organize key system outputs such as SpO_₂_ accuracy, motion artifact handling, and skin tone bias under a single “Outcomes” category rather than listing them individually.

2. Abbreviations should be written out in full only once when first introduced. There is no need to repeat the full terms (abbreviations) in later sections such as the Discussion or Future Directions section.
